# Transitional and Long-Term Care System in Japan and Current Challenges for Stroke Patient Rehabilitation

**DOI:** 10.3389/fneur.2021.711470

**Published:** 2022-01-11

**Authors:** Shoji Kinoshita, Masahiro Abo, Takatsugu Okamoto, Kohei Miyamura

**Affiliations:** ^1^Department of Rehabilitation Medicine, The Jikei University School of Medicine, Tokyo, Japan; ^2^Department of Rehabilitation Medicine, Nishi-Hiroshima Rehabilitation Hospital, Hiroshima, Japan; ^3^Department of Rehabilitation Medicine, Kawakita Rehabilitation Hospital, Tokyo, Japan

**Keywords:** cerebrovascular disease, rehabilitation, long-term care insurance, acute phase, convalescent phase, chronic phase

## Abstract

In Japan, the national medical insurance system and long-term care insurance (LTCI) system cover rehabilitation therapy for patients with acute, convalescent, and chronic stroke. Medical insurance covers early and multidisciplinary rehabilitation therapy during acute phase hospitalizations. Patients requiring assistance in their activities of daily living (ADL) after hospitalization are transferred to kaifukuki (convalescent) rehabilitation wards (KRW), which the medical insurance system has also covered. In these wards, patients can receive intensive and multidisciplinary rehabilitation therapy to improve their ADL and transition to a smooth home discharge. After discharge from these hospitals, elderly patients with stroke can receive outpatient (day-care) rehabilitation and home-based rehabilitation using the LTCI system. The Japanese government has proposed building a community-based integrated care system by 2025 to provide comprehensive medical services, long-term care, preventive care, housing, and livelihood support for patients. This policy aims to promote smooth coordination between medical insurance services and LTCI providers. Accordingly, the medical insurance system allows hospitals to receive additional fees by providing patient information to rehabilitation service providers in the LTCI system. A comprehensive database on acute, convalescent, and chronic phase stroke patients and seamless cooperation between the medical care system and LTCI system is expected to be established in the future. There are only 2,613 board-certified physiatrists in Japan, and many medical schools lack a department for rehabilitation medicine; establishing such a department at each school is encouraged to teach students efficient medical care procedures, to conduct research, and to facilitate the training of personnel in comprehensive stroke rehabilitation.

## Introduction

Historically, stroke has been the number one cause of death among Japanese people, but has shifted to the fourth most common cause in recent years due to decreasing mortality rates, with cancer as the first, heart disease second, and senility third. These numbers reflect fewer deaths due to improved emergency medical services and advances in treatment methods, such as the use of recombinant tissue-type plasminogen activators (rt-PA) and mechanical thrombectomy. However, the overall number of patients with stroke remains high. In Japan, ~220,000 people experience a new stroke and ~290,000 people have recurring strokes annually (1). Endovascular treatment or neurosurgery is administered to 9.1% of patients, and 73% of patients receive rehabilitation. Another characteristic of stroke is that the number of patients affected increases with age. According to the 2019 surveys from the Ministry of Health, Labor, and Welfare, cerebrovascular disease accounted for 16.1% of primary causes, requiring long-term care (2). These diseases are serious problems for public life and health, despite being preventable, to a certain extent, through lifestyle improvements.

**Figure 1:**
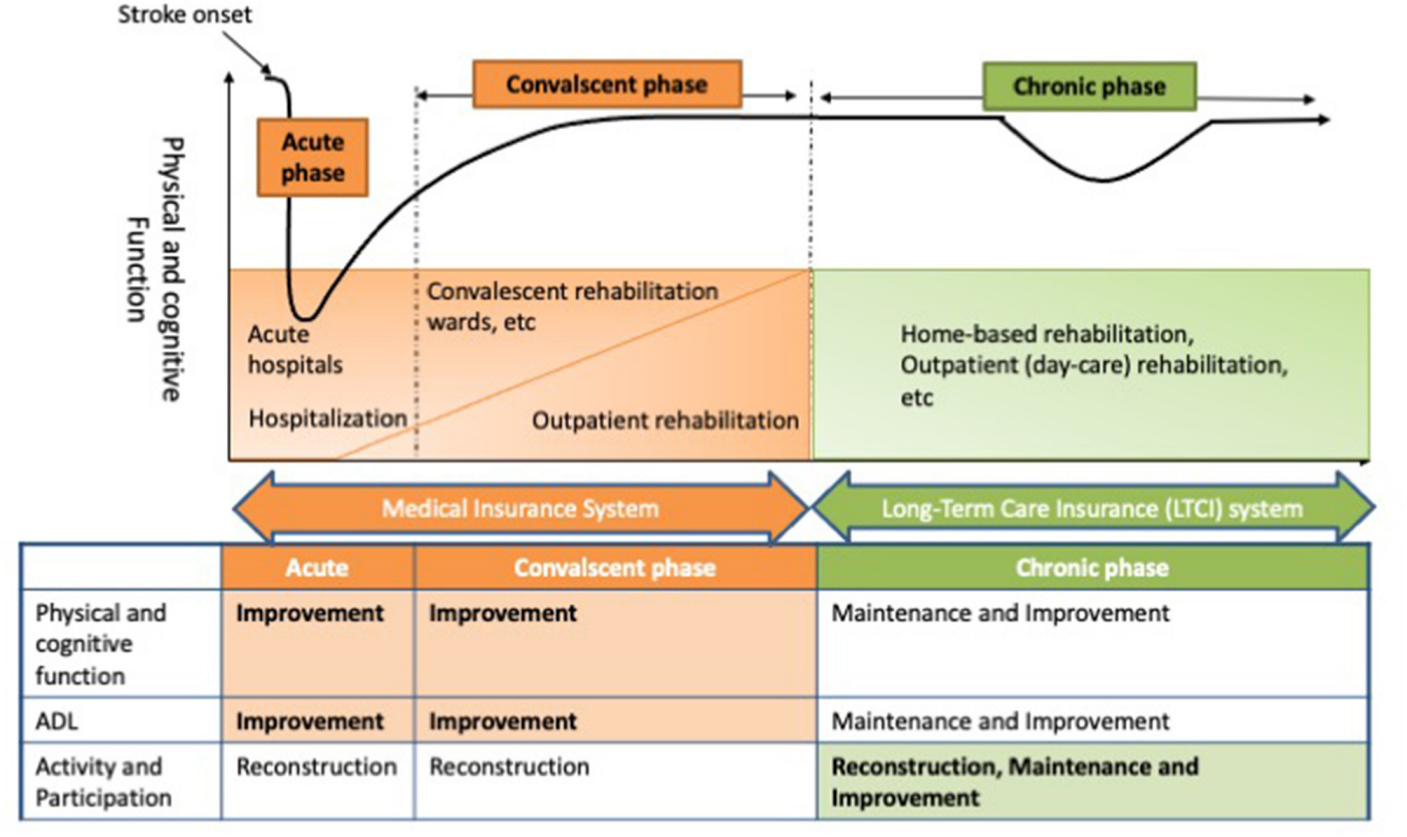
Transitional and long-term care system for rehabilitation of patients with stroke in Japan. In Japan, the medical insurance system covers rehabilitation services for stroke patients in acute and convalescent phases, while the long-term care insurance (LTCI) system covers chronic phase rehabilitation services. In the acute and convalescent phases, the main purpose of rehabilitation is to improve functional recovery and activities of daily living (ADL) of patients with stroke. In the chronic phase, the main aim of rehabilitation services is to maintain physical and cognitive functions and ADL, while improving the engagement of patients with stroke.

The clinical importance of rehabilitation therapy for patients with stroke is well-established. Rehabilitation must be provided in a timely and appropriate manner during acute, convalescent, and chronic phases. It is also important to transition seamlessly between the treatments for each phase to improve and maintain the function of patients with stroke. In Japan, the national medical insurance system and long-term care insurance (LTCI) system were established to provide rehabilitation services for patients with stroke (Figure 1). In fact, Japan's insurance system is unique. Furthermore, the length of hospital stay for patients with acute stroke in Japan is longer than in other countries, and medical costs are higher. It would be useful to introduce the transitional and long-term care system for rehabilitation of patients with stroke in Japan, where the population is aging to consider the adequate transitional and long-term care strategies after stroke in different health care systems.

The purpose of this mini-review is to outline the transitional and long-term care system in Japan and the current challenges for the rehabilitation of patients with stroke at each phase.

## Acute Phase

The main purpose of acute rehabilitation treatment for stroke is to prevent complications associated with immobility, to prevent systemic complications, and to promote functional reorganization of the brain. For this reason, mobilization, basic movement training, gait training, and activities of daily living (ADL) training are implemented as early rehabilitation. In Japan, in recent years, the importance of early rehabilitation for the treatment of acute stroke has been increasingly recognized, and its practice has become widespread. Evidence for these treatments is being collected, which leads to improved knowledge about early rehabilitation therapy (3). In observational studies from Japan (4, 5), very early mobilization was associated with functional recovery of patients with stroke. Initiating rehabilitative care within 24 h of stroke is safe and useful if provided by skilled therapists under physiatrist supervision. Using the Japanese Diagnosis Procedure Combination database, a cohort study analyzed 100,719 consecutive patients with ischemic stroke (6). Their results suggested that early and intensive rehabilitation improved patient ADL during hospitalization. Another study using the Diagnosis Procedure Combination database analyzed 4,266 patients with acute stroke who received intravenous thrombolysis using rt-PA (7). The results showed that a good prognosis at discharge was more likely in the group of patients who started rehabilitation treatment on the day of or after admission. In addition to timing (how early to start), the frequency (how often care is provided) is also important in early rehabilitation treatment (8). Using the Japan Rehabilitation Database, a cohort study of 8,033 patients with the acute cerebrovascular disease showed that high-frequency rehabilitation care (7 days a week for patients with early-onset acute stroke) was associated with better functional recovery (9). In fact, in most cases, rehabilitation was started the day after the onset of symptoms. The percentage of patients receiving rehabilitation 7 days a week was 35.%. The average length of rehabilitation provided per day was 76.7 min. In addition, the average length of stay in the hospital was 29.5 days (10). Based on these results, the medical insurance system can facilitate early rehabilitation therapy for patients with acute stroke in hospitals. Hospitals can charge an additional fee if they provide rehabilitation earlier in the course of the disease. The medical insurance system also assigns rehabilitation staff to intensive care units.

For acute rehabilitation, multidisciplinary cooperation is important for the functional recovery of patients with stroke. For example, early assessment of swallowing function after the stroke onset helps prevent aspiration pneumonia and to promote early oral intake (11). Aoki et al. (12) reported that the activities of a multidisciplinary swallowing team, consisting of nurses, speech therapists, occupational therapists, audiologists, dieticians, dental hygienists, and pharmacists, reduced the incidence of pneumonia in patients with acute cerebrovascular disease. Such a multidisciplinary team, facilitated by the medical insurance system, should be able to assess and manage swallowing function during the acute stroke phase.

## Convalescent Phase

After acute hospitalization, patients who require ADL assistance are transferred to the kaifukuki (convalescent) rehabilitation wards (KRW), which have been covered by the medical insurance system since 2000 (13, 14). Patients with disabling conditions, including stroke, traumatic brain injury, and other neurological diseases, as well as orthopedic diseases, such as hip fractures, are eligible for KRW admission. Under the health insurance system in Japan, rehabilitation therapy (physical, occupational, and speech therapy) is limited to 3 h per day, and the maximum length of stay for patients with stroke in the KRW is limited to 150 days. When rehabilitation goals are met and home or institutional care services are available, the physiatrist can decide to discharge a patient from the KRW. The basic hospitalization fee for the KRW stay is based on the number of medical staff, the provision of rehabilitation on holidays, the percentage of seriously ill patients, the home discharge rate, and motor Functional Independence Measure (FIM) efficiency adjusted by the length of the hospital stay.

The KRW association annual survey reported almost 85,000 KRW beds in 1,500 hospitals in 2019 (15), with an average patient stay length of 67.5 days. The mean patient age was 76.6 years, and 57.8% of patients were female. Stroke was the cause of 36.9% of cases in the KRW. The average time from the stroke onset to the admission to the KRW was 24.2 days. An average of 137.4 min of rehabilitation therapy was provided per day. When the ADL gain of patients with stroke was analyzed in terms of the change in FIM between admission and discharge, the average FIM was determined to be 61.5 points at admission and 84.6 points at discharge, with an average FIM improvement of 23.1 points. Of these, 60.6% of patients were discharged to their homes.

A variety of cutting-edge rehabilitation therapies have been developed and practiced in the KRW. According to a cohort study analyzing 2,325 patients (16), intensive rehabilitation therapy, defined as rehabilitation therapy for more than 15 h per week,was provided for 862 patients (37.1%). Intensive rehabilitation therapy was significantly associated with increased functional gain in elderly patients with stroke in the KRW. Regarding the collaboration between acute care and convalescent rehabilitation, a shorter interval between the stroke onset and admission to the KRW contributes to improved outcomes in patients with ischemic stroke, including ADL, dysphagia, and home discharge rate (17). Prognostic predictions based on a large database have been developed to assess outcomes of patients with stroke (18, 19). Multidisciplinary collaboration from the International Classification of Functioning, Disability, and Health is also practiced according to patient assessment and information sharing (20, 21). Furthermore, innovative rehabilitation using advanced technology, such as robotics, is also becoming more widely developed and practiced (22). Therefore, IRT, collaborations between acute and chronic phase rehabilitation practices, and rehabilitation using innovative techniques are being developed and conducted in KRWs to improve the function of patients with stroke.

## Chronic Phase

Japan has an unprecedented aging population that affects health and long-term care systems. The LTCI system was introduced in Japan in 2000 to address the demands of older people with disabilities based on a user-oriented social insurance system supporting independence (23). Older people with certified LTCI service needs can utilize facility services, in-home services, and community-based services. Following its implementation, the mean length of stay for patients with stroke in rehabilitation hospitals decreased (24). Furthermore, the Japanese government proposed establishing a community-based integrated care system by 2025 to comprehensively provide health care, nursing care, preventive care, housing, and livelihood support for patients (25). This national policy promotes the smooth coordination between medical insurance services and LTCI providers.

There are two main types of rehabilitation services in the LTCI system: home-based rehabilitation and outpatient (day-care) rehabilitation (26, 27). Home-based rehabilitation is provided by rehabilitation staff who visit patient homes. In 2019, there were ~4,600 facilities and 115,000 recipients for home-based rehabilitation (28). Forty min a day, two times a week is a typical service provision for home-based rehabilitation. During outpatient (day-care) rehabilitation, nursing care services, such as meals and bathing, are provided along with hospital-based rehabilitation. In 2019, there were ~8,000 facilities and 600,000 recipients for outpatient (day-care) rehabilitation (29). The outpatient (day-care) rehabilitation is generally provided one time or two times a week for 6–7 h each time. In outpatient (day-care) rehabilitation, rehabilitation to improve physical functions, such as muscle strength training and gait training, is often conducted.

To use LTCI services, a person must be certified as needing long-term care based on their physical and cognitive functions, as well as the status of their nursing care and medical treatment. For certified patients, the system requires that they use LTCI rehabilitation services instead of medical insurance, except when they are hospitalized or in the early stages of their illness. In addition, the medical insurance also covers the outpatient rehabilitation for younger patients with stroke and elderly patients who were not certified as needing long-term care in the chronic phase. Among rehabilitation services after returning home from the KRW, outpatient rehabilitation through medical insurance, outpatient (day-care) rehabilitation through LTCI, and home-based rehabilitation through LTCI each account for ~10% of discharged patients (15).

In the LTCI system, using information and communication technology devices to collaborate with other professions is encouraged. If service providers provide information from medical insurance service centers to LTCI rehabilitation centers, they can charge an additional fee for linking the information using information and communication technology. Furthermore, holding conferences with other professionals within LTCI rehabilitation services is recommended, which also would allow the use of these technologies, such as video teleconferencing systems.

Scientific evidence is necessary to promote high-quality long-term care services. Therefore, the Japanese Ministry of Health, Labor, and Welfare launched a database for LTCI services, the Long-Term Care Information System for Evidence (LIFE), in April 2021. This database stores information on diseases, physical and cognitive functions, rehabilitation goals and interventions, ADL, instrumental ADL, and nutritional status. The purpose of this database is to provide feedback for users and facilities, as well as promote high-quality evidence-based services. Furthermore, by using the LIFE database, service providers can charge another additional fee within the LTCI system. Data from the LIFE database could allow the establishment of evidence for higher quality rehabilitation services for elderly patients, who also suffer from stroke. Although it is possible to link the National Database of medical claims data with the LIFE database in the LTCI system (30), there are no unified outcome measures for rehabilitation with medical insurance and LTCI. Moreover, since there are no standardized codes for individual rehabilitation interventions, it is difficult to clarify effective rehabilitation methods using a database. We hope comprehensive and standardized intervention methods and outcome measures are established for acute, convalescent, and chronic stroke phases.

## Future Perspective

“The Cerebrovascular and Cardiovascular Disease Control Act” was enacted in December 2019 to control national cerebrovascular diseases. “The Japanese National Plan for Promotion of Cardiovascular Disease Control” based on this law set out three goals: prevention of cerebrovascular diseases and dissemination of correct knowledge; improvement of the service delivery system for health, medical care, and welfare; and promotion of research on cerebrovascular diseases (31, 32). By achieving these three goals, the basic plan thus aims to extend healthy life expectancy by 3 or more years and reduce the age-adjusted mortality rate for cerebrovascular diseases by 2040, which is when the elderly population in Japan will reach its peak. This law and basic plan state that coordination between the treatments for acute, convalescent, and chronic phases is important, and that appropriate services related to medical care, nursing care, and welfare should be provided. Since these require governments and prefectures to promote the control of cerebrovascular diseases, it is expected that the transitional and long-term care systems for stroke rehabilitation will be further developed.

Providing appropriate rehabilitation for patients with stroke requires active physiatrist participation. Physiatrists are usually involved in the clinical management of a multidisciplinary rehabilitation team that consists of nurses, physical therapists, occupational therapists, speech therapists, and medical social workers (33). The physiatrist is expected to implement the management of patients with stroke as a leader of the rehabilitation team. Board-certificated physiatrists with sufficient knowledge and experience about stroke rehabilitation are recommended to be the primary care providers for patients with stroke. A retrospective cohort study with the Japan Rehabilitation Database identified that the clinical management provided by board-certified physiatrists in the form of early rehabilitation for patients with acute and convalescent stroke is a significant predictor of a good functional prognosis (10, 34). In Japan, however, stroke rehabilitation is not always provided by a board-certified physiatrist. At some hospitals or facilities, physicians with other specialties lead the rehabilitation team. This is, in part, due to a shortage in the number of board-certified physiatrists. There were only 2,613 in 2021, and many medical schools lack a department for rehabilitation medicine. Establishing such a department in each medical school would help teach students medicine and efficient medical care, enable research, and facilitate the training of personnel in comprehensive stroke rehabilitation.

Innovative neurorehabilitation techniques, such as non-invasive brain stimulation, are effective in functional recovery, primarily in patients with chronic stroke. Our group developed a combined protocol using repetitive transcranial magnetic stimulation (rTMS) and IRT that can effectively improve the function of patients with chronic stroke (35). This protocol is now being implemented in many facilities throughout Japan (36). Although the efficacy of non-invasive brain stimulation for patients with stroke in the acute and convalescent phases is controversial, it may be possible to perform it effectively according to brain condition, such as in the case of interhemispheric inhibition, by using functional brain imaging (37–39). We hope non-invasive brain stimulation and other neurorehabilitation techniques will become more widely implemented in the rehabilitation of patients with stroke for acute and convalescent phases.

## Author Contributions

SK designed concept, drafted the manuscript with important intellectual input from TO and KM, and takes responsibility for whole work from inception to published article. MA provided technical and administrative support and critically assessed the manuscript. All authors have read and approved the final version of the submitted paper.

## Conflict of Interest

The authors declare that the research was conducted in the absence of any commercial or financial relationships that could be construed as a potential conflict of interest.

## Publisher's Note

All claims expressed in this article are solely those of the authors and do not necessarily represent those of their affiliated organizations, or those of the publisher, the editors and the reviewers. Any product that may be evaluated in this article, or claim that may be made by its manufacturer, is not guaranteed or endorsed by the publisher.
